# Improving immunotherapy for the treatment of hepatocellular carcinoma: learning from patients and preclinical models

**DOI:** 10.1038/s44355-025-00018-y

**Published:** 2025-04-03

**Authors:** Romain Desert, Fabio Gianonne, Antonio Saviano, Yujin Hoshida, Mathias Heikenwälder, Pierre Nahon, Thomas F. Baumert

**Affiliations:** 1 Inserm, Institute of Translational Medicine and Liver Disease, Strasbourg, France; 2 University of Strasbourg, Strasbourg, France; 3 Service d’hépato-gastroentérologie, Pôle Hépato-digestif, Institut-Hospitalo-Universitaire, Hôpitaux Universitaires de Strasbourg, Strasbourg, France; 4 Division of Digestive and Liver Diseases, Department of Internal Medicine, University of Texas Southwestern Medical Center, Dallas, USA; 5 Division of Chronic Inflammation and Cancer, German Cancer Research Centre Heidelberg (DKFZ), Heidelberg, Germany; 6 AP-HP, Hôpitaux Universitaires Paris Seine Saint-Denis, Liver Unit, University Sorbonne Paris Nord, UFR SMBH, Bobigny, France; 7 Inserm, UMR-1138 “Functional Genomics of Solid Tumours”, Centre de Recherche des Cordeliers, Université de Paris, Paris, France

## Abstract

Hepatocellular carcinoma (HCC), the third cause of cancer-related deaths worldwide, continues to present significant therapeutic challenges. Despite significant therapeutic advancement in the last decade, the efficacy of systemic treatments for patients with advanced HCC remains unsatisfactory. While the clinical introduction of immune checkpoint inhibitors has improved response rates and overall survival, their clinical success remains still limited. Here we provide a comprehensive review of current and emerging strategies aimed at enhancing the efficacy of immunotherapy for the treatment of HCC. Both clinical studies conducted in patients as well as studies in preclinical models have markedly advanced our understanding of resistance as well as uncovered novel approaches to overcome resistance. Recent progress is paving the way for improved efficacy and safety of novel approaches that will improve the dismal prognosis of patients with advanced HCC.

## Unmet medical need to treat advanced liver cancer

Liver cancer is one of the most commonly diagnosed cancers and the third main cause of cancer-related deaths worldwide, with >900,000 new cases and >800,000 deaths in 2020^1^. Globally, the annual number of new cases of liver cancer is predicted to increase, with 1.4 million people projected to be diagnosed in 2040^2^. Progress in antiviral therapy combined with increased incidence in metabolic diseases leads to an etiological shift from virus-induced to metabolic-associated liver cancer within the next decades^3,4^. Hepatocellular carcinoma (HCC), accounting for 90% of liver cancers, is still difficult to treat. Key reasons include: (1) its development on a background of advanced chronic liver diseases in most cases, (2) its phenotypic, genomic, and molecular heterogeneity, and (3) late-stage detection due to absent or limited clinical symptoms at the early stages^5,6^. Early-stage (Barcelona Clinic Liver Cancer (BCLC) class A) patients can undergo surgical resection, percutaneous ablation, or liver transplantation, which can be considered curative, in well-selected patients, and yield median overall survival (mOS) of >5 and >10 years, respectively^7,8^.

Following the approval of sorafenib around 15 years ago^9^, marked progress has been made in the treatment of advanced HCC. Several novel treatment regimens have been approved^4^. The latest AASLD guidelines^10^ recommend the use of the atezolizumab plus bevacizumab combination; or tremelimumab plus durvalumab, as first line. Sorafenib, Lenvatinib or Durvalumab are alternatives in case of contraindications. Atezolizumab, durvalumab, and tremelimumab are immune checkpoints inhibitors (ICIs), the first two targeting programmed cell death ligand 1 (PD-L1) and the latest cytotoxic T-lymphocyte-associated protein 4 (CTLA-4). The two above mentioned combinations showed improved survival over sorafenib, with a mOS of 19.2 and 16.43 months, respectively^11–13^. However, regardless of the poor survival rate of these patients under treatment, the response rate is limited, with an objective response rate (ORR) of only 25% in both studies and a complete response in only 5% and 2% of patients respectively. Despite a significant improvement in patient survival, the response rate of ICIs remains unsatisfactory. To identify new therapeutic targets, it is critical to unveil the mechanisms of resistance to immunotherapy. Herein, we explore the known mechanism of resistance to ICIs and present the results of pre-clinical models using combination therapy to thwart ICI resistance.

### Immune checkpoint inhibitors

The PD-1/PD-L1 pathway is a crucial regulator of immune tolerance within the tumor microenvironment. PD-1 is a type 1 transmembrane protein belonging to the CD28 immunoglobulin family. Structurally, it comprises a 288-amino-acid-long protein with an extracellular Ig-V-like N-terminal domain, a hydrophobic transmembrane region, and a cytoplasmic tail characterized by two tyrosine residues^14^. These residues form an immune receptor tyrosine-based inhibitory motif (ITIM) and an immune receptor tyrosine-based switch motif (ITSM). It has been elucidated through mutagenetic studies that PD-1’s inhibitory effect on T cells is predominantly mediated via the activated ITSM^15^. PD-1 expression is not limited to T cells but extends to B cells, NK cells, monocytes, dendritic cells (DCs), and various tumor-infiltrating lymphocytes. Importantly, PD-1 is also expressed on regulatory T cells (Tregs), where it inhibits their proliferation and contributes to the immune response’s suppression^16,17^. The PD-1 pathway includes two ligands, PD-L1 and PD-L2, expressed on a range of cells, including pancreatic islet cells, vascular endothelium, and various antigen-presenting cells (APCs). PD-L1’s expression is particularly notable in multiple tumor types, including gastric cancers, melanomas, and HCC, making it a critical target in immunotherapy. PD-L1 binds to PD-1 with a lower affinity compared to PD-L2^18^, yet its widespread expression on tumor cells and significant association with clinical outcomes in different cancers, including HCC^19,20^, made it a better therapeutic target for immunotherapy. Recently, studies discovered the capacity of the co-activator CD80 to form a heterodimer with PD-1 on the surface of APCs, which inhibits its capacity to bind PD-L1 and could explain part of the resistance to therapies targeting immune checkpoints^21^. The engagement of PD-1 with its ligands leads to the inhibition of T-cell activation, which ultimately leads to T-cell exhaustion and tumor progression^22,23^. This process is facilitated by several pro-inflammatory molecules, including interferon-gamma, which play a significant role in upregulating PD-L1 expression^24^. Monoclonal antibodies targeting PD-1 or PD-L1, such as nivolumab, atezolizumab, or durvalumab, effectively prevent the inhibitory signaling and reactivate T-cell-mediated cytotoxicity against tumor cells. They were approved as single agents or as part of combination therapy for the treatment of advanced HCC, either as first-line or second line, and numerous alternative antibodies are currently under clinical trial (see Table 1).

CTLA-4 is a transmembrane protein with a crucial role in regulating the amplitude of T-cell responses during the early priming phase in lymphoid organs. It is actually the first immune checkpoint protein to be discovered^25^, in 1987, and the first one to be successfully targeted with monoclonal antibody in a preclinical model of cancer^26^. Similar to PD-1, CTLA-4 is part of the CD28 immunoglobulin family and binds to B7 ligands (CD80 and CD86) on APCs. The expression of CTLA-4 is not detectable in naive T cells but is upregulated upon T-cell activation and is also expressed in Tregs. CTLA-4 outcompetes CD28 for B7 ligands, resulting in T-cell anergy^27,28^. In APCs, CTLA-4 binding results in reduced CD28 binding and impeded T-cell activation^29^. Moreover, CTLA-4 engagement has been shown to inhibit IL-2 production and T-cell proliferation and to induce cell cycle arrest^30^. This occurs through interaction with PP2A, SHP-2, and PI3K, which transduce downstream signaling that inhibits T-cell receptor signaling, together with the inhibition of other pathways linked to cell proliferation and survival, such as the nuclear factor kappa-light-chain-enhancer of activated B cells (NF-κB), PI3K, and MAP kinase pathways^31–33^. Furthermore, accumulating evidence points to an important role of CTLA-4 signaling in mediating the suppressive functions of Tregs. Anti-CTLA-4 antibodies bind to CTLA-4 molecules with high affinity, leading to Tregs functional blockade, resulting in enhanced T-cell activation and immunological responses to cancer^34^. CTLA-4 inhibitors have shown success in treating metastatic melanomas and are being explored for HCC treatment. CTLA-4 positive T cells and DCs are associated with impaired T-cell functionality^35,36^, and CTLA-4 inhibitors showed some potential in early clinical trials and ex-vivo models^37,38^. Importantly, CTLA-4 inhibition could have some synergetic effects with anti-PD-1^17^. CTLA-4 inhibition by tremelimumab or ipilimumab showed some potential in phase II and phase III clinical trials, either as first or second-line therapy^11,39^. More, a virtual clinical trial of the nivolumab and ipilimumab combinations in >5000 virtual patients confirmed the therapeutic potential of CLTA-4 inhibition in HCC^40^. Numerous clinical trials are ongoing to test CLTA-4 inhibition in combination with other targets for the treatment of HCC (Table 1).

### Approaches to improve the efficacy of ICIs

Among the different strategies to improve the efficacy of ICIs, one of the most promising is neoantigen vaccination. Neoantigens derive from proteins coding gene mutations or non-mutational sources such as RNA alternative splicing or epigenetic remodeling^41^. Tumor-associated antigens (TAA) are exclusive to tumors, they can bind to major histocompatibility complex (MHC) molecules and are recognized by T cells, triggering robust anti-tumor responses. Hence, TAA-based vaccines have the potential to be used alone or in combination with ICIs to trigger a potent antitumour immune response and potentially display some curative capacity. Several clinical trials are ongoing in different cancer types using this technology and some of them showed promising results^42^. Importantly, a recent study from Yarchoan and colleagues used a plasmid encoding a combination of 40 neoantigens in combination with pembrolizumab in 36 patients with advanced HCC, previously treated with multikinase inhibitor. The treatment showed no severe adverse effect, an ORR of ~30%, and ~8% of complete response^43^. These results, especially the complete response rate, are very promising but they need to be validated in larger cohorts.

An alternative strategy to improve the efficacy of ICIs is to combine them with loco-regional therapies (LRTs) such as percutaneous ablation, transarterial chemoembolization (TACE), or radiation therapy^44^. All these procedures lead to tumor cell death, which releases TAA and pro-inflammatory mediators, which in turn may induce tumor-infiltrating immune cell activation and synergize with ICIs^45^. Interestingly, early results suggest promising efficacy: in a recent study with 110 patients receiving atezolizumab plus bevacizumab as first-line treatment for unresectable intermediate-stage HCC, alone or in combination with LRTs, among the 38 patients achieving complete response, 35 had received ICIs together with LRTs and only 3 ICIs alone^46^. Another approach to improve ICIs efficacy uses irreversible electroporation following tumor resection to trigger an immune response in HCC. In several studies, it was shown that this technique induces a protective immune response, which could synergize with ICIs^47,48^. Many combinations involving LRTs and ICIs are under clinical trial^44^ but preliminary results highlight the complexity of accurately assessing the clinical benefits and potential harms of such therapeutic combination, as shown in the Imbrave 050 trial^49^. The extent to which neoadjuvant ICI applied before curative procedures may improve outcomes is also currently tested in clinical trials^50^.

Overall, numerous therapeutic approaches have been tested in combination with ICIs. As an example, in a recent phase II study, we tested a strategy using an antibody targeting phosphatidylserine in combination with pembrolizumab in HCC patients^51^. Phosphatidylserine is a cytoplasmic-facing phospholipid with an important role in the regulation of inflammation and immune escape. Our data suggest a synergistic effects with PD-1 blockade, which needs to be further confirmed.

Apart from these strategies based on a combination of multiple treatments with ICIs, other approaches try to improve ICI treatment by themselves. First, recent insights pointed out how IgG subclass and post-translational modifications impact IgG activity, suggesting potential improvements for PD-1, PD-L1, or CTLA-4 antibodies regarding Fc targets^52^. Notably, different Fc targets of CTLA-4 antibodies play an important role in Tregs inhibition and this could be a way to significantly improve CTLA-4 targeting therapy. Otherwise, a study focused on PD-1 isoforms and identified the Δ42PD-1 as expressed in a subtype of T cells. In different mouse models, they were able to show that antibodies targeting Δ42PD-1 were more efficient than nivolumab^53^. Alternative strategies are based on the delivery of ICI molecules to the tumor. Nanoparticles are submicron-sized structures with a diameter of 150–500 nm^54^. Liu and colleagues used nanoparticles to co-deliver PD-L1 antibody in addition to a sonodynamic agent (Chlorin E6) in a syngeneic mouse model^55^. This led to an efficient delivery of the compound and a synergistic effect of the two approaches boosting the effect of PD-L1 antibody. Similarly, peptide-based nanoparticles were used to co-deliver compounds targeting β-catenin and PD-1 simultaneously in syngeneic mouse models^56^.

### CAR T-cell therapy

Chimeric antigen receptor (CAR) T cells represent a revolutionary approach in cancer immunotherapy^57^. This technology involves genetically engineering a patient’s own T cells to express a CAR that targets a specific antigen present in the tumoral cells. These modified T cells are then expanded in the laboratory and reinfused into the patient, where they can directly recognize and kill cancer cells. Unlike traditional T-cell therapies that rely on the natural ability of T cells to recognize cancer cells, CAR T cells are designed to target tumors with high specificity and potency. In HCC, the application of CAR T-cell therapy holds significant promise. Glypican-3 (GPC3), a protein overexpressed in most HCC cells but not in normal tissues, has been identified as a promising target for CAR T-cell therapy^58–60^. Studies have shown that GPC3-targeted CAR T cells can effectively recognize and eliminate GPC3-positive HCC cells. Several other promising CAR T-cell tumor targets were identified, including alpha-fetoprotein (AFP), Epithelial cell adhesion molecule (EpCAM), Claudin18.2 (CLD18), CD133, and c-MET^61^. Ongoing clinical trials are still on phase 1 and 2 but some preliminary results are encouraging, notably suggesting a promising anti-tumor activity for CD133-directed CAR T cell therapy in advanced HCC^62^.

### Additional immunotherapies

**Fig. 1:**
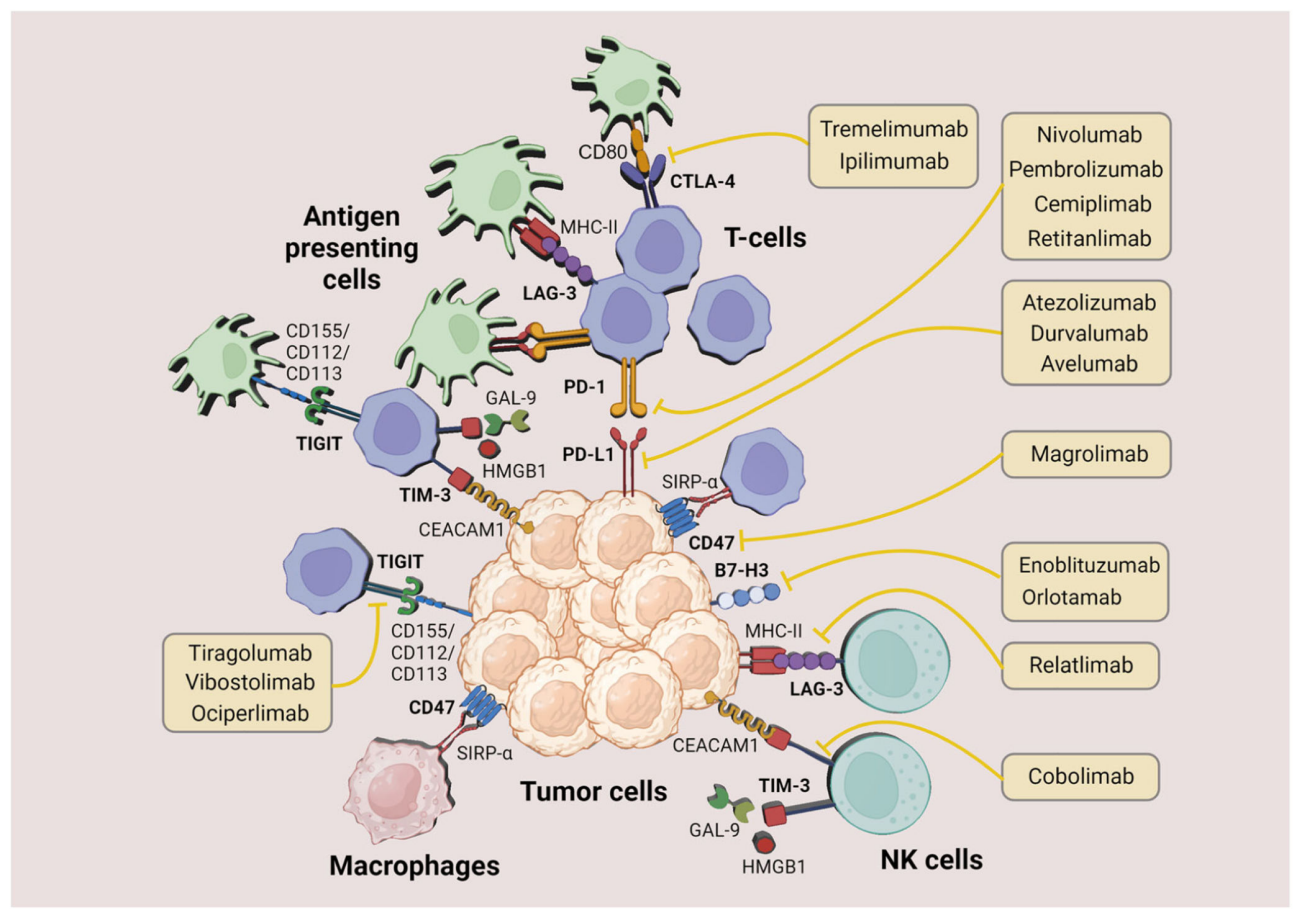
Overview of immune checkpoint inhibitor targets in cancer immunotherapy. This schematic illustrates various immune checkpoint inhibitors (ICIs) and their respective targets within the tumor microenvironment. ICIs include various anti-CTLA-4, anti-PD-1 targeting PD-1 receptors, and anti-PD-L1 antibodies. Magrolimab is depicted targeting CD47 to prevent the ‘don’t eat me’ signal on cancer cells, while TIGIT inhibitors interact with TIGIT receptors on T cells. Other ICIs, such as Enoblituzumab and Orlotamab, are shown to target unspecified antigens, Relatlimab binds LAG3 on T cells, and Cobolimab targets TIM3. The figure underscores the complex interplay between the immune system and cancer cells and highlights the multiplicity of potential therapeutic targets for ICIs.

In the past 10 years, the development of novel immunotherapies has been enormously successful, resulting in the identification of novel immune checkpoints such as T-cell immunoglobulin and ITIM domain (TIGIT)^63^, T-cell immunoglobulin and mucin-domain containing-3 (TIM3)^64^, lymphocyte activation gene-3 (LAG3)^65^, CD47^66^ and B7 homolog 3 protein (B7-H3)^67^, among others (for a more extensive list, see^68^). Each of these proteins has distinct ligands and suppress T-cell function through several mechanisms to inhibit T-cell response (Fig. 1). Yet for the moment, phase III clinical trials are missing to evaluate the therapeutic potential of these new targets in HCC patients (Table 1).

### Hypothetical mechanisms and immune cells involved in resistance to checkpoint inhibitors

The advent of ICIs has marked a significant breakthrough in the treatment of various malignancies, with substantial clinical efficacy across several cancer types^69^. Particularly in Hodgkin’s disease and desmoplastic melanoma, immune checkpoint inhibitor therapy has achieved outstanding success, with ORR > 70% with the nivolumab plus pembrolizumab combination; but for most cancers (including HCC), treatment responses remain unsatisfactory. Moreover, ~10% of HCC patients under ICIs experience faster and more aggressive tumor progression than expected (known as hyperprogressive disease) for which the mechanisms of action are poorly understood^70,71^. Resistance to immunotherapy can occur in two different ways. Either patients are primary non-responders, or resistance is acquired after a period of documented response to therapy^72^. Of note, challenges remain in defining responders and non-responders, given the heterogeneity in patterns of response to ICI, such as spatial or temporal heterogeneity, manifesting within a given patient as mixed responses^73^.

**Fig. 2:**
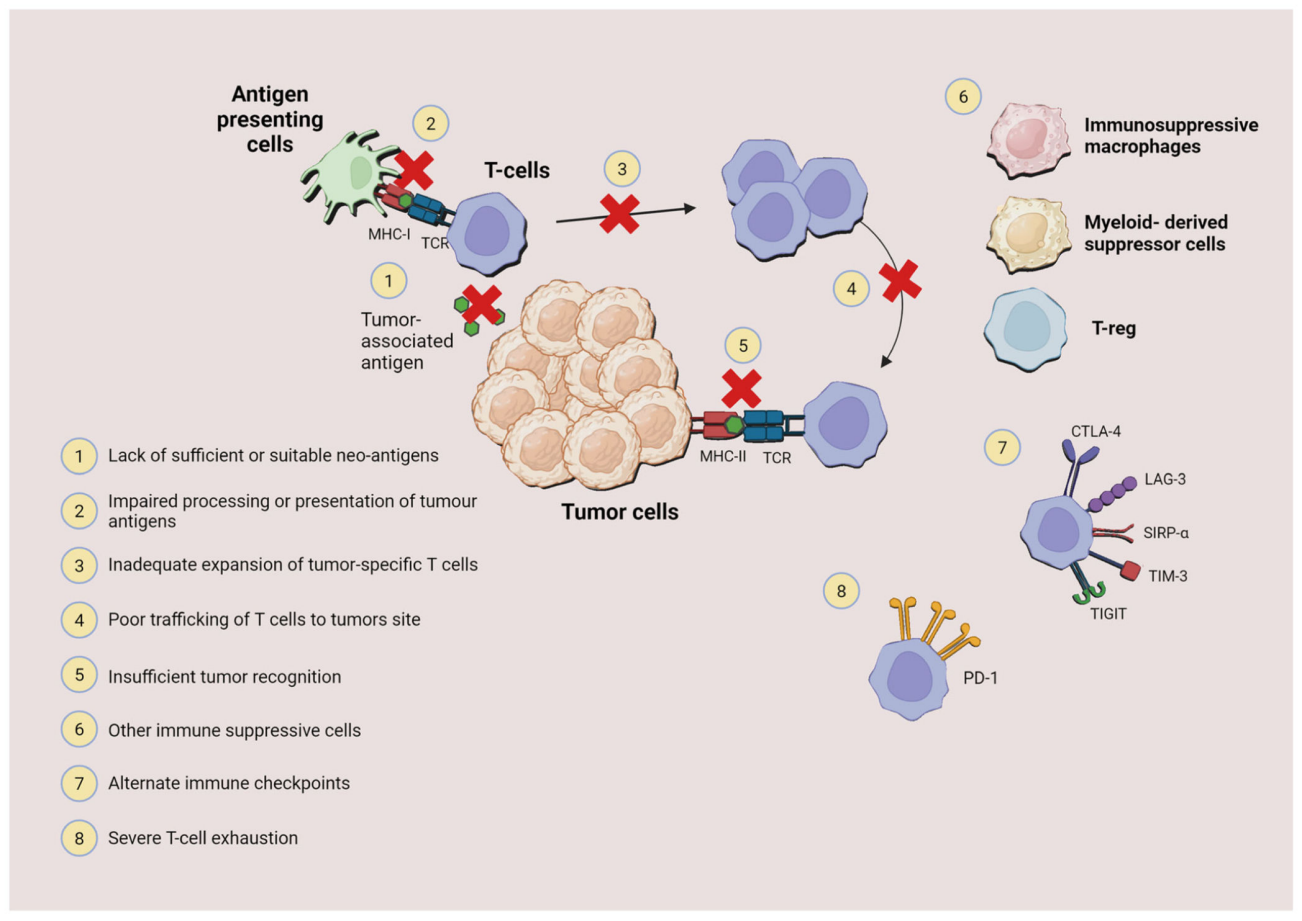
Mechanisms of resistance to immune checkpoint inhibitors in hepatocellular carcinoma. Illustration of the various mechanisms through which HCC cells may develop resistance to immune checkpoint inhibitors (ICIs). 1) Mutation or altered expression of antigen-presenting molecules on tumor cells leading to reduced T-cell recognition. 2) Impaired antigen presentation. 3) Alterations in the tumor microenvironment, or secretion of immunosuppressive cytokines by tumor cells. 4) Impaired T-cell phenotype, or a physical barrier repressing T-cell infiltration. 5) Altered tumor recognition by T cells. 6) Recruitment of immunosuppressive macrophages, myeloid-derived suppressor cells (MDSCs), or regulatory T cells (Tregs) to the tumor microenvironment, suppressing the activation and proliferation of T cells. 7) Upregulation of alternate immune checkpoints in tumor cells, rendering standard ICIs ineffective. 8) T-cell exhaustion is characterized by the overexpression of multiple inhibitory receptors, diminishing the immune response. The figure underscores the complexity of the tumor-immune microenvironment and the multifaceted nature of immune resistance in HCC, highlighting the need for multi-targeted approaches.

While the exact mechanisms of innate and/or acquired resistance to ICI remain to be fully unveiled, several mechanisms have been hypothesized based on the known mechanisms of action of these therapies and preclinical studies^74^. These include: decreased neoantigen expression, impaired antigen recognition, ineffective antigen presentation, insufficient priming and activation of tumor-specific T cells, inadequate expansion of T cells or lack of co-stimulation, poor trafficking of the activated effector T cells to the tumor site, insufficient cancer cell recognition by T cells, presence of T-cell inhibitory factors or other T-cell inhibitory immune cells in the tumor microenvironment (TME) (Fig. 2)^75^. In HCC, the liver’s unique immune microenvironment, characterized by a high prevalence of immunosuppressive cells like Kupffer cells and hepatic stellate cells, also contributes to resistance by creating an immunotolerant environment that diminishes the efficacy of ICIs^76^. The mechanisms at stake in ICI resistance are complex and involve the crosstalk between many cell populations and subpopulations within the TME.

One of the mediators of this process is tumor-associated macrophages (TAMs). In theory, macrophages are dichotomized into M1 and M2 phenotypes, with M1 macrophages exerting pro-inflammatory effects, while M2 macrophages contribute to immunosuppression and tissue repair. However, these subclasses have been difficult to identify in vivo and single-cell RNAseq analysis identified a more complex pattern of distribution of TAMs^77^. Yet the prevalence of immunosuppressive “M2-like” macrophages, characterized by markers CD163 and CD206, is associated with an aggressive HCC phenotype, advanced tumor stage, and poor survival outcomes^78^. The presence of these immunosuppressive TAMs, especially at the tumor margin, is linked to adverse clinical features like vascular invasion, tumor multiplicity, and fibrous capsule formation^79^. Even if the role of the TAMs in ICI resistance has not been fully established in HCC, TAMs remain on the top of the suspects list. Regarding the potential mechanisms, studies in other cancers suggest that “M2-like” macrophages inhibit cytotoxic T cell through IL-10 secretion, and promote immunosuppressive phenotype in other macrophages, NK cells, and Tregs by IL-6, VEGF, and CSF-1^80–82^.

Tregs are another cell population with a crucial role in immune exhaustion and resistance to ICIs. Naturally occurring FoxP3 + CD25 + CTLA-4 + CD4+ Tregs are indispensable for immunological self-tolerance. They come from the thymus and derive from the differentiation of T cells with intermediate T-cell receptor (TCR) affinity for self-peptide/MHC ligands while T cells with low TCR affinity differentiate into naive conventional T cells^17,83^. Tregs are thus able to recognize self-antigens, which may also be tumor-associated neoantigens. In addition to these thymus-derived Tregs, different sets of FoxP3 positive or negative immunosuppressive Tregs can differentiate in the tumor tissue from conventional T cells; the mechanisms at stake are not fully understood but involve IL-2 and TGF-β^84^. In general, high Treg/CD8 + T-cell ratios in tumors correlate with tumor progression and poor survival^85,86^. Tregs inhibit antigen presentation on APCs via CTLA-4 and have other immunosuppressive functions through the cell surface molecules, CD25, CD39, and CD73, and the cytokines IL-10, IL-35, and TGF-β^17^. PD-1 also inhibits Tregs activity as PD-1 blockade can result in increased Tregs activation^87^. PD-1 positive Tregs in tumors may undermine PD-1 blockade immunotherapy, as shown by the positive correlation between PD-1 positive Treg and hyperprogressive disease in gastric cancer^88^.

In addition to TAMs and Tregs, tumor-associated neutrophils (TANs) can also be immunosuppressive. Under activation by cancer-associated fibroblasts (CAFs) or HCC cells, they can express PD-L1 and release anti-inflammatory molecules, such as IL8, TNF, and CCl2, which inhibits T-cell activation^89^, or promotes Tregs recruitment^90^. Myeloid-derived suppressor cells (MDSCs) are a heterogeneous subset of myeloid cells that have been shown to inhibit T-cell responses in cancer and HCC. MDSCs can inhibit CD8 + T-cell proliferation and their accumulation is associated with poor survival rates in HCC patients^91^. More, MDSCs can promote Tregs and repress NK cell cytotoxicity by NKp30-dependent cell contact^92,93^.

### Mechanisms of resistance to checkpoint inhibitors: learning from patients

In addition to cell-based studies to characterize the mechanisms of resistance to ICIs, an alternative strategy is to analyze tumor samples to compare the molecular, genetic, and clinical features of patient responders with non-responders. In an elegant study by Zhu and colleagues, tumor samples from 358 HCC patients treated with atezolizumab plus bevacizumab were enrolled from the GO30140 phase 1b and IMbrave 150 phase 3 trials^94^. The authors discovered that pre-existing immunity, marked by dense intratumoural CD8 + T cells, was linked to improved clinical outcomes. Conversely, reduced benefits were associated with a high ratio of Tregs to effector T cells and the expression of oncofetal genes, as well as with β-catenin mutation. The study also indicated that improved outcomes from the combination therapy, as opposed to atezolizumab alone, were linked to high expression of VEGF Receptor 2, Tregs, and myeloid inflammation signatures. These findings, validated through analyses of pre- and post-treatment biopsies, in situ analyses, and in vivo mouse models, suggest that the anti-VEGF component might synergize with anti-PD-L1 therapy by targeting angiogenesis, Tregs proliferation, and myeloid cell inflammation. The study also analyzed the potential effect of the tumor mutational burden (TMB) on the response to ICIs and showed some inconclusive results. In theory, elevated TMB, often caused by DNA repair deficiency, leads to high level of neoantigens, which increases the immune response and the capacity to target ICIs^95,96^. However, a recent study in lung and colon cancer showed that high mutational burden can at the same time leads to strong tumor heterogeneity, which jeopardizes the efficacy of ICIs^97^. In turn, TMB are insufficient to predict response to ICIs and the presence of clonal neoantigens appears to be a better alternative. Overall, the study by Zhu and colleagues confirmed the potential of analyzing the association between treatment response and the patient molecular profiles. Their study also identified a gene signature predicting progression-free survival after atezolizumab-bevacizumab initiation (called the atezolizumabbevacizumab response signature). Later, this signature was validated in some independent cohorts by Zeng and colleagues and the signature was even predicted based on the analysis of patient pathology slides using artificial intelligence-based algorithms^98^. This technic could be used in the future to predict ICIs outcome in patients and better assign future treatments.

Another study from Liu and colleagues performed spatial transcriptomics analysis in 11 HCC samples treated with atezolizumab plus bevacizumab (6 non-responders, 5 responders) and highlighted a histological structure called “immune barrier”, only in non-responders^99^. This structure, composed of TAMs expressing osteopontin interacting with CAFs could act as a physical barrier to T-cell infiltration. Osteopontin is highly expressed in HCC cells and is a well-established predictor of tumor progression and poor outcome^100,101^. Osteopontin secretion by tumoural cells activates macrophages migration through CD44 signaling^102^; these activated macrophages further secrete CSF-1, which eventually induces PD-L1 expression in HCC cells^82^. Moreover, single-cell analysis of HCC patients identified ostepontin as a marker of a subpopulation of cancer-specific macrophages^103^, which is consistent with the data from Liu and colleagues on a potential immune barrier in HCC^99^. The concept of the immune barrier can be linked with the concept of “fibrous nest”^104^, or “fibrotic niche”, an histological structure observed in a subset of HCC patients, that we recently characterized by matrisome analysis^105^. We showed that this phenotype is associated with cancer-specific extracellular matrix remodeling, signatures of Wnt and TGFβ signaling, and immune evasion. All this is consistent with the immune classification of HCC, initiated by Sia and recently refined by Montironi and colleagues^106,107^. For long HCCs have been classified based on transcriptomic profile, and works from different teams yields to different classifications^108–113^, ultimately integrated into a system where HCC are divided into two groups: proliferative, associated with poor outcome and *TP53* mutation and non-proliferative, associated with better outcome and β-catenin (*CTNNB1*) mutation. In the immune-based classification, patients are first divided into two groups: inflamed and non-inflamed, based on the expression of a series of markers of immune activation. Within the inflamed group, patients are subdivided into immune-active, immune-exhausted, and immune-like. Patients expressing the Hoshida’s S1/Wnt/TGFβ signature^111^ are found mostly within the immune active and immune exhausted groups, confirming the link between activation of Wnt/β-Catenin and TGF-β signaling pathways with immune evasion. Both Wnt and TGF-β signaling are known to promote an immunosuppressive TME in HCC. Wnt signaling leads to T-cell exclusion and resistance to ICI therapy through a decrease in chemokines secretion^114^. Elevated TGF-β signaling increase PD-L1 expression and stimulate Tregs expansion, thus disrupting the effectiveness of both anti-PD-L1 and anti-CTLA-4 antibodies^115–117^. In other cancer types, the decrease in TGF-β-induced collagen deposition has been shown to reactivate adaptative immune response and to improve the efficacy of ICIs, confirming the link between intratumour fibrosis and resistance to immunotherapy^118–120^. Overall, it is likely that TGF-β secretion by tumor cells in immune exhausted patients induce an immunosuppressive TME and activate CAFs, which form a collagen-based fibrous nest, acting as an immune barrier for infiltrating T cells.

Within the non-inflamed group, patients are subdivided into intermediate and immune-excluded. Patients with *CTNNB1* mutation, which represent ~30% of HCCs^5^, are mostly associated with the immune-excluded subclass, consistent with their known resistance to ICIs^121,122^. Even if the mechanisms of immune exclusion driven by *CTNNB1* mutation are not fully understood, a model based on hydrodynamic tail vein injection (HDTV) of a mutated *CTNNB1* showed that *CTNNB1* mutation impairs the recruitment of DCs and subsequent T-cell activation, mediated by a reduction in CCL5^122^. Another study showed that the immunosuppressive phenotype in *CTNNB1* mutated HCC was mediated by TNFRSF19-mediated repression of cytokines secretion^123^. Of note, HCC with *CTNNB1* mutation was not resistant to ICIs in an HDTV-based mouse model^94^. Overall, the association between the immune-based molecular classification and response to immunotherapy is appealing but needs to be confirmed in large prospective patient cohorts. This could lead to improving our comprehension of the molecular mechanisms of resistances in different patient subsets and to the rise of personalized medicine in advanced HCCs.

In addition to these approaches based on patient clustering, alternative signaling pathways activated in HCC have been shown to have some immunomodulatory features and appear as interesting therapeutic targets. The EGFR-P38 MAPK axis in HCC cells also enhances immunosuppression by upregulating PD-L1 expression and suppressing HLA-I expression^124^. The loss of PTEN, a tumor suppressor gene frequently mutated in HCC^125^, can activate the PI3K signaling pathway, leading to decreased T-cell infiltration and increased immunosuppression. This loss impairs the stimulation of pathways like type I interferon and NF-κB, contributing to tumor progression due to the immunosuppressive TME^126,127^. All these proteins identified as immunomodulators could be targeted, which could improve the efficacy of ICIs in HCC.

### Combination therapy: learning from preclinical models

The advancement of ICI therapy in cancer has been greatly facilitated by the recent evolution of preclinical models. Traditionally, the study of liver cancer relied on cell-based models and in vivo xenografts in immunodeficient mice. These models, while invaluable in understanding cancer biology and evaluating the therapeutic potential of chemotherapies, fell short in elucidating the complex interactions between tumor cells and the immune system, which is critical to assessing ICI efficacy. The growing need to accurately assess the potential of ICIs necessitated a shift toward models that embody the intricacies of the tumor-immune microenvironment. Coculture systems mark a step forward, allowing direct interactions between tumor cells and some immune cells in a controlled environment. In theory, they could offer insights into the immunomodulatory effects of ICIs, but these systems are limited by their lack of architectural complexity and the absence of a full physiological immune system. Patient-derived organoids, represent a more physiologically relevant model, preserving the three-dimensional architecture and cellular heterogeneity of tumors^128^. They serve as a bridge between traditional cell culture and in vivo studies, providing a more accurate platform for drug screening. Their limitation is the absence of a competent immune system, which can be bypassed by combining coculture systems and organoids^129,130^. Another alternative is the use of patient-derived spheroids, cultured in patients-derived serum^131^. This system provides all the advantages of the previous one but displays a complete immune system, which can be used to assess the effects of ICIs. Using such a system, we discovered a previously undiscovered immunomodulatory capacity of a therapy targeting Claudin-1, a protein expressed on the cell surface of HCC cells. Treatment with CLDN1-specific antibodies has been shown to markedly inhibit HCC growth by inhibiting pro-carcinogenic signaling and reprogramming the TME^132^. Syngeneic mouse models, wherein tumor cells from a mouse strain are implanted into a genetically identical host, preserve an intact immune system, thus providing an invaluable context for studying ICIs^133^. They offer insights into the antitumour immune response and the development of resistance. However, these models often lack the genetic diversity and complexity of human HCC, potentially oversimplifying the immune TME. Chemically induced HCC models in mice replicate the multistage development of liver cancer, providing a spectrum of diseases from dysplasia to carcinoma^134–136^. They are valuable for studying the natural evolution of HCC and the immune responses at different stages. Yet, the long latency and the variability in tumor development are significant drawbacks, posing challenges for timely and uniform study designs. Diethylnitrosamine, in particular, the most widely used model of chemically induced HCC, apart from its long time to tumor development (8–12 months), generates tumors with genetic mutations that poorly recapitulate the mutational landscape of human HCC^137^ and harboring poor immune infiltration^138^. This model is often associated with chemically induced or diet-induced liver fibrosis, either with carbon tetrachloride or Western diet in order to accelerate the tumor development and to get closer to the physiopathology of HCC development^134,139^. Similarly, genetically engineered mouse models with transgenic overexpression of an oncogene and/or ablation of an anti-oncogene, offer the possibility to evaluate the effect of ICIs in immunocompetent animal, but by definition, they can only recapitulate a narrow spectrum of genetic alterations, which can be problematic for the translation to human^140^. The HDTV technique is a relatively recent innovation, enabling the study of gene function in liver carcinogenesis through the rapid introduction of genetic material into hepatocytes^141^. This method can mimic the genetic alterations seen in human HCC and allows for the study of tumor-immune interactions in an intact immune system. By the choice of the oncogenes overexpression, or the anti-oncogenes deletion, they also provide a variety of cancer phenotypes, associated with different immune profiles, which mimics, to a certain extend, the variability of human HCC^142,143^. More, models using some plasmid combinations of oncogenes are sensitive to ICIs, while others are resistant^143,144^. Humanized mice, engineered to possess a human immune system, provide a critical platform for evaluating the efficacy of ICIs in a context that closely mimics human immune responses^145^. They are particularly useful for studying ICIs targeting human-specific antigens. The cons include the high cost, the need for specialized facilities, and the fact that the reconstituted human immune system may not fully recapitulate the diversity and functionality of its natural counterpart.

Table 2 provide an extended list of the preclinical studies that used immunocompetent mouse models to study the possibility of combination therapy of ICIs with other drugs. They were performed using a variety of mouse models including syngeneic, HDTV, chemically induced, or transgenic mouse models. The most widely used model was the subcutaneous injection of Hepa1-6 cells. This HCC mouse cell line originates from the BW7756 cells, generated by Jackson laboratory from C57L/J mice in the 60’s and first used in research in 1971^146^, before it was in vitro subcloned into different Hepa1 variants^147^ and that the Hepa1-6 was identified^148^. Since then, the Hepa1-6 cell line has emerged as a reliable tool to easily and cost-effectively generate fast-growing tumors, both in C57L and C57/BL6, upon subcutaneous or orthotopic (i.e., intrahepatic) injection. Tumors from this model partly respond to ICIs but are sensitive to a broad spectrum of combination therapies, which improve the efficacy of ICIs. Recently, Zabransky and colleagues performed cytometry by time of flight to profile the TME of different syngeneic mouse models of HCC, including Hepa1-6^149^. They highlighted a rather important CD8 T-cell infiltration as well as PD-L1 expression in Hepa1-6 orthotopic model. They also performed integration with human data and found only a few HCC samples with immune profiles matching the one of Hepa1-6, which questions the translationality of data using this model. Of note, while Hepa1-6 subcutaneously injected were sensitive to anti-PD-1 therapy, it was not the case for orthotopic injection in this study, underlying the importance of orthotopic models to better recapitulate the characteristics of the liver TME for ICIs evaluation.

As shown in Table 2, a broad spectrum of therapeutic targets has shown a potential to improve the efficacy of ICIs, which underlines the wide possibilities for refining current treatments. First, some studies have been using ICIs in combination with standard chemotherapy such as oxaliplatin, or with multikinase inhibitors already approved as single agents for the treatment of HCC (sorafenib or Lenvatinib). These studies suggest some potential in combining ICIs with standard treatments, as recently performed for the treatment of cholangiocarcinoma, where ICIs have been approved in combination with chemotherapy^12,150^. Alternatively, studies tried some molecules from traditional medicine like Scutellarin^151^ or YIV-906^152^, which highlight the potential of drugs already known to have anticancer properties to improve ICIs-based therapy. Studies like the one from Bao and colleagues also confirm that vascular normalization strategies can enhance ICI efficacy^153^. By a combination of VEGF/VEGFR2 inhibitors with anti-PD-1 therapy, they showed a significant increase in CD8 T-cell infiltration, a significant reduction in tumor size, and improved survival rates in treated mice. Another study used regorafenib, a multikinase inhibitor known for its anti-angiogenic properties, with PD-1 blockade and found that regorafenib not only hindered angiogenesis but also altered the TME to make it more receptive to ICIs^154^. As expected, a majority of studies showing improvement of ICIs used immunomodulatory agents, like antibodies targeting TIGIT^144^, CXCR2^134^, CSF-1-R^82,155^, osteopontin^99^ or triggering receptor expressed on myeloid cells-1 (TREM-1)^156^. Others used nonsteroidal anti-inflammatory drugs (meloxicam)^157^ or interferon-α^158,159^, or approaches closer to a vaccination strategy, by the use of components triggering an immunization in the mouse, by using polyinosinic-polycytidylic acid^160^, DCs vaccine^161^ or AFP immunization^162^. Another strategy is to focus on the metabolic pathways associated with HCC and highlight that modulating these networks has the potential to improve immunotherapy. For example, Luo and colleagues identified Prmt5, encoding protein arginine N-methyltransferase 5, as a key metabolic modulator of MYC-induced HCC; and by disrupting this pathway they could enhance the response to anti-PD-1 therapy^163^. Other drugs targeting liver metabolisms showed some potential, like cholecystokinina antagonist^164^ or peroxisome proliferator-activated receptor gamma antagonist^165^. More analysis focused on novel molecular and genetic targets with unknown roles in liver cancer, such as N6-methyladenosine reader YTHDF1, Poly(ADP-ribose) glycohydrolase, or Cholecystokinin-B Receptor. These three new therapeutic targets were shown to be targetable and to synergize with ICIs^164,166,167^. Collectively, all these studies demonstrate the potential of combining ICIs with a variety of agents targeting different aspects of tumor biology and the TME. Each approach offers unique insights into enhancing the efficacy of immunotherapy in HCC, emphasizing the need for multifaceted and tailored treatment strategies to overcome the complex nature of the disease.

## Conclusion and future avenues

Emerging strategies in the treatment of HCC through ICIs reveal the multifaceted nature of cancer therapy. The development and utilization of immunocompetent preclinical models are instrumental in understanding the complex interactions between tumor cells and the immune system, but significant challenges arise when translating these findings into clinical practice. Syngeneic mouse models, genetically engineered mice, chemically induced, and diet-induced models only partially capture the diversity of the tumor-immune microenvironment from human HCC. Bridging the gap between preclinical research and clinical application remains a significant challenge in cancer research. Nevertheless, the exploitation of combination therapies using these models targeting the TME from multiple angles has revealed important new information to improve cancer therapy. Investigating the molecular diversity of HCC has uncovered new therapeutic targets. The potential of CAR T-cell therapy remains still to be determined for HCC but may open a perspective for a more personalized treatment approach. As we move forward, the integration of these diverse therapeutic strategies promises to enhance the efficacy of current therapies. The combination of discovery and innovation will bring the field closer to more effective, personalized treatments for HCC patients—a key unmet medical need. The collective effort of clinicians and scientists will undoubtedly pave the way for improved efficacy and safety and, ultimately, improved prognosis for patients.

## Figures and Tables

**Table 1:** Published and ongoing phase III randomized control trials using immune checkpoint inhibitors in unresectable or metastatic hepatocellular carcinoma

Trial	Year publication or estimated completion	Tested therapy	Targets	Population	Result/main outcome	FDA Approved
*Published*						
First line (advanced stage)						
IMbrave 150	2020	Atezolizumab + Bevacizumab vs Sorafenib	PD-L1 +VEGF	Locally advanced or metastatic HCC, Child A	improved OS and PFS vs Sorafenib	Yes
ORIENT-32	2021	Sintilimab + Bevacizumab vs Sorafenib	PD-1 +VEGF	Cyto/Histopathological diagnosis, Chinese population, HBV associated HCC, Child A/B7	Improved OS and PFS vs Sorafenib	No
COSMIC-312	2022	Atezolizumab + Cabozantinib vs Sorafenib	PD-L1 + multikinase	HCC on cirrhosis, not other curative or loco-regional therapies, Child A	Longer PFS in the Atezolizumab/ Cabozantinib group, no differences in OS	No
HIMALAYA	2022	Durvalumab + Tremelimumab or Durvalumab vs Sorafenib	PD-L1 + CTLA-4	Histopathological confirmation, Child A	Improved OS than sorafenib. Durvalumab only was not inferior to Sorafenib	Yes
CheckMate 459	2022	Nivolumab vs sorafenib	PD-1	Histopathological diagnosis, possible previous surgery or loco-regional treatments, Child A	No differences in OS	Yes
CARES-310	2023	Camrelizumab + Rivoceranib vs Sorafenib	PD-1 +VEGFR2	Cyto/Histopathological diagnosis, Child A	Benefit in PFS and OS compared with sorafenib	No
LEAP-002	2023	Pembrolizumab + Lenvatinib vs Lenvatinib + placebo	PD-1 + multikinase	No loco-regional or curative possibilities, Child A	No difference in OS and PFS versus Lenvatinib plus placebo	No
RATIONALE-301	2023	Tislelizumab vs Sorafenib	PD-1	Histopathological confirmation, Child A	Similar OS, a higher objective response rate in Tislelizumab, longer PFS with sorafenib	No
*Second line or more (advanced stage)*						
KEYNOTE-240	2020	Pembrolizumab vs placebo	PD-1	Prior Sorafenib	Favorable risk-to-benefit ratio for pembrolizumab, no statistical difference for OS and PFS	Yes
KEYNOTE-394	2022	Pembrolizumab vs placebo	PD-1	Prior Sorafenib or oxaliplatin-based CT, patients from Asia	Pembrolizumab improved OS and PFS	Yes
*Currently ongoing*						
Adjuvant therapy						
JUPITER-04 (NCT 03859128)	2023	Toripalimab vs placebo	PD-1	Adjuvant therapy in resected HCC with high risk of recurrence	RFS	
RANT (NCT 05277675)	2023	Tislelizumab (or Sintilimab) + Lenvatinib (or Bevacizumab) + RFA vs RFA	PD-1 +	Neoadjuvant therapy, no possibility of resection, BCLC-0 or A, Child A/B7	RFS	
PREVENT-2 (NCT05910970)	2024	Tislelizumab + Lenvatinib vs Tislelizumab	PD-1 + multikinase	Adjuvant therapy in HCC at high risk of recurrence after resection or ablation, Child A-B7	RFS	
CheckMate 9DX (NCT 03383458)	2025	Nivolumab vs placebo	PD-1	Adjuvant therapy in resected or ablated HCC with high risk of recurrence, Child A	RFS	
EMERALD-2 (NCT03847428)	2025	Durvalumab + Bevacizumab vs Durvalumab + placebo vs placebo	PD-L1 +VEGF	Adjuvant therapy in resected or ablated HCC with high risk of recurrence	RFS	
IMbrave 050 (NCT04102098)	2027	Atezolizumab + Bevacizumab vs surveillance	PD-L1 +VEGF	Adjuvant therapy in HCC at high risk of recurrence after resection or ablation	RFS	
KEYNOTE-937 (NCT 03867084)	2029	Pembrolizumab vs placebo	PD-1	Adjuvant therapy in cases with complete radiologic response after resection or ablation	RFS, OS	
Intermediate stage						
CheckMate 74 W (NCT04340193)	2024	Nivolumab + Ipilimumab vs Nivolumab vs placebo, all in combination with TACE	PD-1 + CTLA-4	Eligible patients for TACE	Safety	
NCT04229355	2023	Sorafenib vs Lenvatinib vs PD-1 Inhibitor, all in combination with DEB-TACE	PD-1 + multikinase	Unresectable HCC, Child A	PFS	
ABC-HCC (NCT04803994)	2025	Atezolizumab + Bevacizumab vs TACE	PD-L1 +VEGF	No curative options, Child A, possible previous resection/ ablation/ TACE	Time to failure	
TACE-3 (NCT04268888)	2026	Nivolumab vs placebo, all in combination with TACE/TAE	PD-1	Child A, no indication for resection/ transplantation	os	
EMERALD-1 (NCT03778957)	2026	Durvalumab + TACE* followed by Durvalumab, Durvalumab + TACE* followed by Durvalumab + Bevacizumab, TACE only	PD-L1	Child A or B7, not candidate for surgery	PFS	
REPLACE (NCT04777851)	2027	Pembrolizumab + Regorafenib vs TACE or TARE	PD-1 + multikinase	Child A, ALBI 1—2, no contraindication to intra-arterial treatments	PFS	
LEAP-012 (NCT04246177)	2029	Pembrolizumab + Lenvatinib vs placebo, all in combination with TACE	PD-1	Incurable/ Non-metastatic HCC	PFS	
First line (advanced stage)						
NCT03605706	2023	Camrelizumab + FOLFOX4 vs FOLFOX4 + placebo	PD-1	Child A/B7	OS	
CheckMate 9DW (NCT04039607)	2026	Nivolumab + Ipilimumab vs standard of care (Sorafenib or Lenvatinib)	PD-1 + CTLA-4	Child A, no brain metastasis	OS	
TRIPLET (NCT05665348)	2026	Atezolizumab + Bevacizumab with or without Ipilimumab	PD-L1 + CTLA-4	No curative or loco-regional options	Reponse to treatment, OS	
SKYSCRAPER-14 (NCT05904886)	2026	Atezolizumab + Bevacizumab with or without Tiragolumab	PD-L1 +TIGIT	Locally advanced or metastatic HCC, Child A	PFS	
Second line or more (advanced stage)						
IMbrave251 (NCT04770896)	2025	Atezolizumab + standard of care (Sorafenib or Lenvatinib) vs standard of care alone	PD-L1 + multikinase	Previously treated with Atezolizumab and Bevacizumab, Child A/B7	OS	

* or DEB-TACE.

*DEB-TACE* drug-eluting bead transarterial chemoembolization, *OS* overall survival, *PFS* progression-free survival, *RFA* radiofrequency ablation, *RFS* recurrence-free survival, *TACE* transarterial chemoembolization, *TAE* transarterial embolization, *TARE* transarterial radioembolization.

**Table 2:** Summary of preclinical models of combination therapy to improve ICIs for HCC

Model type	Model	Tumor site	Primary target	Combination therapy	Year	Ref.
Syngeneic	Hepa1-6	Liver	PD-1	Scutellarin	2023	151
Syngeneic	Hepa1-6	Sub-cut.	PD-L1	SIRT2 inhibitor (AGK2)	2023	168
Chemically induced	DEN	Liver	PD-1	Blockmir CD5-2	2023	138
Syngeneic	Hepa1-6	Sub-cut.	PD-1	SPP1 antibody	2023	99
Hydrodynamic tail vein injection	c-MYC/Mcl1	Liver	PD-1 + PD-L1	AFP immunization	2023	162
Syngeneic	RIL-175	Sub-cut.	PD-1	proglumide (CCK-receptor antagonist)	2023	164
Transgenic mice	C57BL/6J-TG(ALB1HBV)44BRI/J	Liver	PD-L1	5-AZA	2023	169
Syngeneic	RIL-175	Liver	PD-1	Ythdf1 siRNA	2023	166
Syngeneic	Hepa1-6	Sub-cut.	PD-1	PPT1 inhibitor (DC661)	2023	170
Syngeneic + Hydrodynamic tail vein injection	Hepa1-6 + RIL-175 + N-Ras;c-Myc	Sub-cut. + Liver	PD-L1	PPARγ antagonist (T0070907)	2023	165
Syngeneic	Hep55-1C	Sub-cut. + Liver	PD-1	Immunogenic peptides vaccination	2023	171
Hydrodynamic tail vein injection	Trp53KO/CMycOE	Liver	PD-1	Sorafenib	2023	143
Syngeneic	Hepa1-6	Sub-cut.	PD-1	DDK inhibitor (XL413)	2023	172
Hydrodynamic tail vein injection	CTNNB1N90;Trp53KO	Liver	PD-1	CD36 inhibitor (SSO)	2023	173
Syngeneic	Hepa1-6	Sub-cut.	PD-1	Meloxicam	2022	157
Chemically induced	DEN + ALIOS Diet	Liver	PD-1	CXCR2 inhibitor (AZD5069)	2022	134
Syngeneic	Hepa1-6	Sub-cut.	PD-1	HIF inhibitor 32-134D	2022	174
Syngeneic	Hepa1-6	Sub-cut.	PD-L1	YAP inhibitor (Verteporfin)	2022	175
Syngeneic	Hepa1-6	Sub-cut.	PD-1	PKCα inhibitor(Gö6976) + CSF-1-R inhibitor (BLZ945) + lenvatinib	2022	155
Hydrodynamic tail vein injection	CTNNB1N90;c-MetOE	Liver	PD-1	FAK inhibitor (VS4718)	2022	176
Syngeneic	Hepa1-6 + H22	Liver	PD-1	PARG inhibitor (COH34)	2022	167
Syngeneic	Hepa1-6	CCl4- treated liver	PD-L1	Simvastatin	2022	177
Syngeneic	Hepa1-6	Sub-cut. + Liver	PD-1	pegylated interferon-a	2022	159
Syngeneic	H22	Sub-cut.	PD-1	Combretastatin A4 nanoparticule + VEGFR2inhibitor(DC101)	2021	153
Transgenic + syngeneic	tetO-MYC transgenic + H22 + Hepa1.6	Liver + Sub-cut.	PD-1	PRMT5 inhibitor (GSK3326595)	2021	163
Syngeneic	Hepa1-6 + Hep-53.4	Sub-cut. + Liver	PD-1	Lenvatinib	2021	178
Syngeneic	Hepa1-6	Liver	PD-L1	HDAC8 inhibitor (PCI-34051)	2021	179
Syngeneic	Hepa1-6	Sub-cut.	PD-1	YIV-906 (PHY906)	2021	152
Syngeneic	Hepa1-6	Sub-cut.	PD-1	Lenvatinib	2021	180
Hydrodynamic tail vein injection	Trp53KO;c-MycOE	Liver	PD-1	TIGIT	2020	144
Syngeneic fibrotic	Hepa1-6 in CCl4 treated	Liver	PD-L1	BET inhibitor (i-BET762)	2020	181
Syngeneic	HCA-1 + RIL-175	Liver	PD-1	VEGFR2 inhibitor (DC101)	2020	182
Syngeneic	RIL-175	Liver	PD-1	Regorafenib	2020	154
Syngeneic	Hep55.1 C	Liver	PD-1	Dendritic cells vaccine	2020	161
Syngeneic	H22	Sub-cut.	PD-1	Oxaliplatine	2020	183
Syngeneic	Hepa1-6	Liver	PD-1	photothermal agent DiR + interferon genes agonist (vadimezan)	2019	158
Hydrodynamic tail vein injection	N-RasOE;c-MycOE	Liver	PD-L1	PolyIC	2019	160
Syngeneic	Hepa1-6	Liver	PD-L1	TREM-1 inhibitor (GF9)	2019	156
Syngeneic	Hepa1-6 + H22	Sub-cut. + Liver	PD-L1	CSF-1R inhibitor (PLX3397)	2019	82

## Data Availability

No datasets were generated or analyzed during the current study.

## Abbreviations

APCs: antigen-presenting cells
B7-H3 B7: homolog 3 protein
BCLC: Barcelona clinic liver cancer
CAFs: cancer-associated fibroblasts
CAR: Chimeric antigen receptor
CTLA-4: cytotoxic T-lymphocyte-associated protein 4
DCs: dendritic cells
HCC: Hepatocellular carcinoma
HDTV: hydrodynamic tail vein injection
ICIs: immune checkpoints inhibitors
ITSM: immune receptor tyrosine-based switch motif
ITIM: immune receptor tyrosine-based inhibitory motif
LAG3: lymphocyte activation gene-3
LRTs: loco-regional therapies
MDSCs: myeloid-derived suppressor cells
MHC: major histocompatibility complex
mOS: median overall survival
NF-κB: nuclear factor kappa-light-chain-enhancer of activated B cells
ORR: objective response rate
PD-L1: programmed cell death ligand 1
TAA: Tumor-associated antigens
TACE: transarterial chemoembolization
TAMs: tumor-associated macrophages
TANs: tumor-associated neutrophils
TCR: T-cell receptor
TIGIT: T-cell immunoglobulin and ITIM domain
TIM3: T-cell immunoglobulin and mucin-domain containing-3
Tregs: regulatory T cells
TME: tumor microenvironment
TMB: tumor mutational burden

## References

[1] Sung H (2021). Global Cancer Statistics 2020: GLOBOCAN estimates of incidence and mortality worldwide for 36 cancers in 185 countries. CA Cancer J Clin.

[2] Rumgay H (2022). Global burden of primary liver cancer in 2020 and predictions to 2040. J Hepatol.

[3] Estes C (2018). Modeling NAFLD disease burden in China, France, Germany, Italy, Japan, Spain, United Kingdom, and United States for the period 2016–2030. J Hepatol.

[4] Singal AG, Kanwal F, Llovet JM (2023). Global trends in hepatocellular carcinoma epidemiology: implications for screening, prevention and therapy. Nat Rev Clin Oncol.

[5] Llovet JM (2021). Hepatocellular carcinoma. Nat Rev Dis Prim.

[6] Sia D, Villanueva A, Friedman SL, Llovet JM (2017). Liver cancer cell of origin, molecular class, and effects on patient prognosis. Gastroenterology.

[7] Tabrizian P (2022). Ten-year outcomes of liver transplant and downstaging for hepatocellular carcinoma. JAMA Surg.

[8] Reveron-Thornton RF (2022). Global and regional long-term survival following resection for HCC in the recent decade: a meta-analysis of 110 studies. Hepatol Commun.

[9] Llovet JM (2008). Sorafenib in advanced hepatocellular carcinoma. N Engl J Med.

[10] Singal AG (2023). AASLD practice guidance on prevention, diagnosis, and treatment of hepatocellular carcinoma. Hepatology.

[11] Abou-Alfa GK (2022). Tremelimumab plus durvalumab in unresectable hepatocellular carcinoma. NEJM Evid.

[12] Finn RS (2020). Atezolizumab plus bevacizumab in unresectable hepatocellular carcinoma. N Engl J Med.

[13] Cheng AL (2022). Updated efficacy and safety data from IMbrave150: atezolizumab plus bevacizumab vs. sorafenib for unresectable hepatocellular carcinoma. J Hepatol.

[14] Kornepati AVR, Vadlamudi RK, Curiel TJ (2022). Programmed death ligand 1 signals in cancer cells. Nat Rev Cancer.

[15] Riley JL (2009). PD-1 signaling in primary T cells. Immunol Rev.

[16] Li Q (2023). Antibody-based cancer immunotherapy by targeting regulatory T cells. Front Oncol.

[17] Tay C, Tanaka A, Sakaguchi S (2023). Tumour-infiltrating regulatory T cells as targets of cancer immunotherapy. Cancer Cell.

[18] Cheng X (2013). Structure and interactions of the human programmed cell death 1 receptor. J Biol Chem.

[19] Gao Q (2009). Overexpression of PD-L1 significantly associates with tumour aggressiveness and postoperative recurrence in human hepatocellular carcinoma. Clin Cancer Res.

[20] Zeng T (2023). Expression pattern of PD-1/PD-L1 in primary liver cancer with clinical correlation. Liver Int.

[21] Sugiura D (2019). Restriction of PD-1 function by cis-PD-L1/CD80 interactions is required for optimal T cell responses. Science.

[22] Li Q, Han J, Yang Y, Chen Y (2022). PD-1/PD-L1 checkpoint inhibitors in advanced hepatocellular carcinoma immunotherapy. Front Immunol.

[23] Wang J (2021). Clinical outcomes and influencing factors of PD-1/PD-L1 in hepatocellular carcinoma. Oncol Lett.

[24] Muhlbauer M (2006). PD-L1 is induced in hepatocytes by viral infection and by interferon-alpha and -gamma and mediates T cell apoptosis. J Hepatol.

[25] Brunet JF (1987). A new member of the immunoglobulin superfamily-CTLA-4. Nature.

[26] Leach DR, Krummel MF, Allison JP (1996). Enhancement of antitumour immunity by CTLA-4 blockade. Science.

[27] Rudd CE, Taylor A, Schneider H (2009). CD28 and CTLA-4 coreceptor expression and signal transduction. Immunol Rev.

[28] Yang W (2023). A novel CTLA-4 blocking strategy based on nanobody enhances the activity of dendritic cell vaccine-stimulated antitumour cytotoxic T lymphocytes. Cell Death Dis.

[29] Krummel MF, Allison JP (1995). CD28 and CTLA-4 have opposing effects on the response of T cells to stimulation. J Exp Med.

[30] Hannani D (2015). Anticancer immunotherapy by CTLA-4 blockade: obligatory contribution of IL-2 receptors and negative prognostic impact of soluble CD25. Cell Res.

[31] Hu H, Rudd CE, Schneider H (2001). Src kinases Fyn and Lck facilitate the accumulation of phosphorylated CTLA-4 and its association with PI-3 kinase in intracellular compartments of T-cells. Biochem Biophys Res Commun.

[32] Schneider H, Smith X, Liu H, Bismuth G, Rudd CE (2008). CTLA-4 disrupts ZAP70 microcluster formation with reduced T cell/APC dwell times and calcium mobilization. Eur J Immunol.

[33] Kim GR, Choi JM (2022). Current understanding of cytotoxic T lymphocyte antigen-4 (CTLA-4) Signaling in T-cell biology and disease therapy. Mol Cells.

[34] Rowshanravan B, Halliday N, Sansom DM (2018). CTLA-4: a moving target in immunotherapy. Blood.

[35] Han Y (2014). Human CD14+ CTLA-4+ regulatory dendritic cells suppress T-cell response by cytotoxic T-lymphocyte antigen-4-dependent IL-10 and indoleamine-2,3-dioxygenase production in hepatocellular carcinoma. Hepatology.

[36] Kalathil S, Lugade AA, Miller A, Iyer R, Thanavala Y (2013). Higher frequencies of GARP(+)CTLA-4(+)Foxp3(+) T regulatory cells and myeloid-derived suppressor cells in hepatocellular carcinoma patients are associated with impaired T-cell functionality. Cancer Res.

[37] Pedroza-Gonzalez A (2015). GITR engagement in combination with CTLA-4 blockade completely abrogates immunosuppression mediated by human liver tumour-derived regulatory T cells ex vivo. Oncoimmunology.

[38] Sangro B (2013). A clinical trial of CTLA-4 blockade with tremelimumab in patients with hepatocellular carcinoma and chronic hepatitis C. J Hepatol.

[39] Yau T (2020). Efficacy and safety of nivolumab plus ipilimumab in patients with advanced hepatocellular carcinoma previously treated with sorafenib: the CheckMate 040 randomized clinical trial. JAMA Oncol.

[40] Sové RJ (2022). Virtual clinical trials of anti-PD-1 and anti-CTLA-4 immunotherapy in advanced hepatocellular carcinoma using a quantitative systems pharmacology model. J Immunother Cancer.

[41] Hu Z, Guo X, Li Z, Meng Z, Huang S (2024). The neoantigens derived from transposable elements - a hidden treasure for cancer immunotherapy. Biochim Biophys Acta Rev Cancer.

[42] Guasp P, Reiche C, Sethna Z, Balachandran VP (2024). RNA vaccines for cancer: principles to practice. Cancer Cell.

[43] Yarchoan M (2024). Personalized neoantigen vaccine and pembrolizumab in advanced hepatocellular carcinoma: a phase 1/2 trial. Nat Med.

[44] Llovet JM (2021). Locoregional therapies in the era of molecular and immune treatments for hepatocellular carcinoma. Nat Rev Gastroenterol Hepatol.

[45] McLaughlin M (2020). Inflammatory microenvironment remodelling by tumour cells after radiotherapy. Nat Rev Cancer.

[46] Kudo M (2023). Achievement of complete response and drug-free status by atezolizumab plus bevacizumab combined with or without curative conversion in patients with transarterial chemoembolization-unsuitable, intermediate-stage hepatocellular carcinoma: a multicenter proof-of-concept study. Liver Cancer.

[47] Dai Z (2021). Irreversible electroporation induces CD8(+) T cell immune response against post-ablation hepatocellular carcinoma growth. Cancer Lett.

[48] Qian J (2020). Blocking exposed PD-L1 elicited by nanosecond pulsed electric field reverses dysfunction of CD8(+) T cells in liver cancer. Cancer Lett.

[49] Qin S (2023). Atezolizumab plus bevacizumab versus active surveillance in patients with resected or ablated high-risk hepatocellular carcinoma (IMbrave050): a randomised, open-label, multicentre, phase 3 trial. Lancet.

[50] Llovet JM (2024). Adjuvant and neoadjuvant immunotherapies in hepatocellular carcinoma. Nat Rev Clin Oncol.

[51] Hsiehchen D (2024). The phosphatidylserine targeting antibody bavituximab plus pembrolizumab in unresectable hepatocellular carcinoma: a phase 2 trial. Nat Commun.

[52] Nimmerjahn F, Vidarsson G, Cragg MS (2023). Effect of posttranslational modifications and subclass on IgG activity: from immunity to immunotherapy. Nat Immunol.

[53] Tan Z (2023). Isoformic PD-1-mediated immunosuppression underlies resistance to PD-1 blockade in hepatocellular carcinoma patients. Gut.

[54] Pavelic K (2023). Nanoparticles in medicine: current status in cancer treatment. Int J Mol Sci.

[55] Liu Y (2022). Nanobubble-based anti-hepatocellular carcinoma therapy combining immune check inhibitors and sonodynamic therapy. Nanoscale Adv.

[56] Zhou Z (2023). Targeting beta-catenin and PD-L1 simultaneously by a racemic supramolecular peptide for the potent immunotherapy of hepatocellular carcinoma. Theranostics.

[57] Labanieh L, Mackall CL (2023). CAR immune cells: design principles, resistance and the next generation. Nature.

[58] Jiang Z (2016). Anti-GPC3-CAR T cells suppress the growth of tumour cells in patient-derived xenografts of hepatocellular carcinoma. Front Immunol.

[59] Wu X (2019). Combined antitumour effects of sorafenib and GPC3-CAR T cells in mouse models of hepatocellular carcinoma. Mol Ther.

[60] Li D (2020). Persistent polyfunctional chimeric antigen receptor T cells that target glypican 3 eliminate orthotopic hepatocellular carcinomas in mice. Gastroenterology.

[61] Ozer M, Goksu SY, Akagunduz B, George A, Sahin I (2023). Adoptive cell therapy in hepatocellular carcinoma: a review of clinical trials. Cancers.

[62] Dai HR (2020). Efficacy and biomarker analysis of CD133-directed CAR T cells in advanced hepatocellular carcinoma: a single-arm, open-label, phase II trial. Oncoimmunology.

[63] Jantz-Naeem N (2023). TIGIT signaling and its influence on T cell metabolism and immune cell function in the tumour microenvironment. Front Oncol.

[64] Tian T, Li Z (2021). Targeting Tim-3 in cancer with resistance to PD-1/PD-L1 blockade. Front Oncol.

[65] Huo JL, Wang YT, Fu WJ, Lu N, Liu ZS (2022). The promising immune checkpoint LAG-3 in cancer immunotherapy: from basic research to clinical application. Front Immunol.

[66] Son J (2022). Inhibition of the CD47-SIRPalpha axis for cancer therapy: a systematic review and meta-analysis of emerging clinical data. Front Immunol.

[67] Zhou WT, Jin WL (2021). B7-H3/CD276: an emerging cancer immunotherapy. Front Immunol.

[68] Dutta S, Ganguly A, Chatterjee K, Spada S, Mukherjee S (2023). Targets of immune escape mechanisms in cancer: basis for development and evolution of cancer immune checkpoint inhibitors. Biology.

[69] Ribas A, Wolchok JD (2018). Cancer immunotherapy using checkpoint blockade. Science.

[70] Pinter M, Scheiner B, Pinato DJ (2023). Immune checkpoint inhibitors in hepatocellular carcinoma: emerging challenges in clinical practice. Lancet Gastroenterol Hepatol.

[71] Wei Z, Zhang Y (2022). Immune cells in hyperprogressive disease under immune checkpoint-based immunotherapy. Cells.

[72] Sharma P, Hu-Lieskovan S, Wargo JA, Ribas A (2017). Primary, adaptive, and acquired resistance to cancer immunotherapy. Cell.

[73] Michielin O, Lalani AK, Robert C, Sharma P, Peters S (2022). Defining unique clinical hallmarks for immune checkpoint inhibitor-based therapies. J Immunother Cancer.

[74] Jenkins RW, Barbie DA, Flaherty KT (2018). Mechanisms of resistance to immune checkpoint inhibitors. Br J Cancer.

[75] Xie Q, Zhang P, Wang Y, Mei W, Zeng C (2022). Overcoming resistance to immune checkpoint inhibitors in hepatocellular carcinoma: challenges and opportunities. Front Oncol.

[76] Ringelhan M, Pfister D, O’Connor T, Pikarsky E, Heikenwalder M (2018). The immunology of hepatocellular carcinoma. Nat Immunol.

[77] Roszer T (2015). Understanding the mysterious M2 macrophage through activation markers and effector mechanisms. Mediat Inflamm.

[78] Dong P (2016). CD86(+)/CD206(+), diametrically polarized tumour-associated macrophages, predict hepatocellular carcinoma patient prognosis. Int J Mol Sci.

[79] Ding T (2009). High tumour-infiltrating macrophage density predicts poor prognosis in patients with primary hepatocellular carcinoma after resection. Hum Pathol.

[80] Donne R, Lujambio A (2023). The liver cancer immune microenvironment: therapeutic implications for hepatocellular carcinoma. Hepatology.

[81] Cassetta L, Pollard JW (2023). A timeline of tumour-associated macrophage biology. Nat Rev Cancer.

[82] Zhu Y (2019). Disruption of tumour-associated macrophage trafficking by the osteopontin-induced colony-stimulating factor-1 signalling sensitises hepatocellular carcinoma to anti-PD-L1 blockade. Gut.

[83] Klein L, Kyewski B, Allen PM, Hogquist KA (2014). Positive and negative selection of the T cell repertoire: what thymocytes see (and don’t see). Nat Rev Immunol.

[84] Bayati F (2020). The therapeutic potential of regulatory T cells: challenges and opportunities. Front Immunol.

[85] Tu JF (2016). Regulatory T cells, especially ICOS(+) FOXP3(+) regulatory T cells, are increased in the hepatocellular carcinoma microenvironment and predict reduced survival. Sci Rep.

[86] Schoenberg MB (2021). The predictive value of tumour infiltrating leukocytes in hepatocellular carcinoma: a systematic review and meta-analysis. Eur J Surg Oncol.

[87] Vick SC, Kolupaev OV, Perou CM, Serody JS (2021). Anti-PD-1 checkpoint therapy can promote the function and survival of regulatory T cells. J Immunol.

[88] Kamada T (2019). PD-1(+) regulatory T cells amplified by PD-1 blockade promote hyperprogression of cancer. Proc Natl Acad Sci USA.

[89] Cheng Y (2018). Cancer-associated fibroblasts induce PDL1+ neutrophils through the IL6-STAT3 pathway that foster immune suppression in hepatocellular carcinoma. Cell Death Dis.

[90] Zhou SL (2016). Tumour-associated neutrophils recruit macrophages and t-regulatory cells to promote progression of hepatocellular carcinoma and resistance to sorafenib. Gastroenterology.

[91] Zhou J (2018). Hepatoma-intrinsic CCRK inhibition diminishes myeloid-derived suppressor cell immunosuppression and enhances immune-checkpoint blockade efficacy. Gut.

[92] Hoechst B (2009). Myeloid derived suppressor cells inhibit natural killer cells in patients with hepatocellular carcinoma via the NKp30 receptor. Hepatology.

[93] Hoechst B (2008). A new population of myeloid-derived suppressor cells in hepatocellular carcinoma patients induces CD4(+)CD25(+) Foxp3(+) T cells. Gastroenterology.

[94] Zhu AX (2022). Molecular correlates of clinical response and resistance to atezolizumab in combination with bevacizumab in advanced hepatocellular carcinoma. Nat Med.

[95] Miao D (2018). Genomic correlates of response to immune checkpoint blockade in microsatellite-stable solid tumours. Nat Genet.

[96] McGranahan N (2016). Clonal neoantigens elicit T cell immunoreactivity and sensitivity to immune checkpoint blockade. Science.

[97] Westcott PMK (2023). Mismatch repair deficiency is not sufficient to elicit tumour immunogenicity. Nat Genet.

[98] Zeng Q (2023). Artificial intelligence-based pathology as a biomarker of sensitivity to atezolizumab-bevacizumab in patients with hepatocellular carcinoma: a multicentre retrospective study. Lancet Oncol.

[99] Liu Y (2023). Identification of a tumour immune barrier in the HCC microenvironment that determines the efficacy of immunotherapy. J Hepatol.

[100] Song Z (2021). Osteopontin takes center stage in chronic liver disease. Hepatology.

[101] Desert R (2022). Role of hepatocyte-derived osteopontin in liver carcinogenesis. Hepatol Commun.

[102] Jiang X (2021). Lipid-injured hepatocytes release sOPN to improve macrophage migration via CD44 engagement and pFak-NFkappaB signaling. Cytokine.

[103] Sharma A (2020). Onco-fetal reprogramming of endothelial cells drives immunosuppressive macrophages in hepatocellular carcinoma. Cell.

[104] Desert R (2016). “Fibrous nests” in human hepatocellular carcinoma express a Wnt-induced gene signature associated with poor clinical outcome. Int J Biochem Cell Biol.

[105] Desert R (2023). Hepatocellular carcinomas, exhibiting intratumour fibrosis, express cancer-specific extracellular matrix remodeling and WNT/TGFB signatures, associated with poor outcome. Hepatology.

[106] Sia D (2017). Identification of an immune-specific class of hepatocellular carcinoma, based on molecular features. Gastroenterology.

[107] Montironi C (2023). Inflamed and non-inflamed classes of HCC: a revised immunogenomic classification. Gut.

[108] Lee JS (2004). Classification and prediction of survival in hepatocellular carcinoma by gene expression profiling. Hepatology.

[109] Boyault S (2007). Transcriptome classification of HCC is related to gene alterations and to new therapeutic targets. Hepatology.

[110] Chiang DY (2008). Focal gains of VEGFA and molecular classification of hepatocellular carcinoma. Cancer Res.

[111] Hoshida Y (2009). Integrative transcriptome analysis reveals common molecular subclasses of human hepatocellular carcinoma. Cancer Res.

[112] Desert R (2017). Human hepatocellular carcinomas with a periportal phenotype have the lowest potential for early recurrence after curative resection. Hepatology.

[113] Cancer Genome Atlas Research Network. Electronic address wbe, Cancer Genome Atlas Research N (2017). Comprehensive and integrative genomic characterization of hepatocellular carcinoma. Cell.

[114] Morita M (2023). Role of beta-catenin activation in the tumour immune microenvironment and immunotherapy of hepatocellular carcinoma. Cancers.

[115] David CJ, Massague J (2018). Contextual determinants of TGFbeta action in development, immunity and cancer. Nat Rev Mol Cell Biol.

[116] Flavell RA, Sanjabi S, Wrzesinski SH, Licona-Limon P (2010). The polarization of immune cells in the tumour environment by TGFbeta. Nat Rev Immunol.

[117] Gonzalez-Sanchez E (2021). The TGF-beta pathway: a pharmacological target in hepatocellular carcinoma?. Cancers.

[118] Li L (2021). Laminin gamma2-mediating T cell exclusion attenuates response to anti-PD-1 therapy. Sci Adv.

[119] Horn LA (2022). Remodeling the tumour microenvironment via blockade of LAIR-1 and TGF-beta signaling enables PD-L1-mediated tumour eradication. J Clin Invest.

[120] Zhang D (2022). Enhancing CRISPR/Cas gene editing through modulating cellular mechanical properties for cancer therapy. Nat Nanotechnol.

[121] Harding JJ (2019). Prospective genotyping of hepatocellular carcinoma: clinical implications of next-generation sequencing for matching patients to targeted and immune therapies. Clin Cancer Res.

[122] Ruiz de Galarreta (2019). Beta-catenin activation promotes immune escape and resistance to anti-PD-1 therapy in hepatocellular carcinoma. Cancer Discov.

[123] Wong AM (2022). Unique molecular characteristics of NAFLD-associated liver cancer accentuate beta-catenin/TNFRSF19-mediated immune evasion. J Hepatol.

[124] Liu Z (2021). The EGFR-P38 MAPK axis up-regulates PD-L1 through miR-675-5p and down-regulates HLA-ABC via hexokinase-2 in hepatocellular carcinoma cells. Cancer Commun.

[125] Schulze K (2015). Exome sequencing of hepatocellular carcinomas identifies new mutational signatures and potential therapeutic targets. Nat Genet.

[126] Vidotto T (2020). Emerging role of PTEN loss in evasion of the immune response to tumours. Br J Cancer.

[127] Lin Z (2021). PTEN loss correlates with T cell exclusion across human cancers. BMC Cancer.

[128] LeSavage BL, Suhar RA, Broguiere N, Lutolf MP, Heilshorn SC (2022). Next-generation cancer organoids. Nat Mater.

[129] Yuan J, Li X, Yu S (2022). Cancer organoid co-culture model system: novel approach to guide precision medicine. Front Immunol.

[130] Zhou Z (2023). Harnessing 3D in vitro systems to model immune responses to solid tumours: a step towards improving and creating personalized immunotherapies. Nat Rev Immunol.

[131] Crouchet E (2021). A human liver cell-based system modeling a clinical prognostic liver signature for therapeutic discovery. Nat Commun.

[132] Roehlen N (2023). Treatment of HCC with claudin-1-specific antibodies suppresses carcinogenic signaling and reprograms the tumour microenvironment. J Hepatol.

[133] Blidisel A (2021). Experimental models of hepatocellular carcinoma-a preclinical perspective. Cancers.

[134] Leslie J (2022). CXCR2 inhibition enables NASH-HCC immunotherapy. Gut.

[135] Moeini A (2019). An immune gene expression signature associated with development of human hepatocellular carcinoma identifies mice that respond to chemopreventive agents. Gastroenterology.

[136] Koelsch N (2023). The crosstalking immune cells network creates a collective function beyond the function of each cellular constituent during the progression of hepatocellular carcinoma. Sci Rep.

[137] Connor F (2018). Mutational landscape of a chemically-induced mouse model of liver cancer. J Hepatol.

[138] Liu K (2023). Novel miRNA-based drug CD5-2 reduces liver tumour growth in diethylnitrosamine-treated mice by normalizing tumour vasculature and altering immune infiltrate. Front Immunol.

[139] Filliol A (2022). Opposing roles of hepatic stellate cell subpopulations in hepatocarcinogenesis. Nature.

[140] Liu S (2022). Mouse models of hepatocellular carcinoma: classification, advancement, and application. Front Oncol.

[141] Sebestyen MG (2006). Mechanism of plasmid delivery by hydrodynamic tail vein injection. I. Hepatocyte uptake of various molecules. J Gene Med.

[142] Molina-Sanchez P (2020). Cooperation between distinct cancer driver genes underlies intertumour heterogeneity in hepatocellular carcinoma. Gastroenterology.

[143] Yuen VW (2023). Using mouse liver cancer models based on somatic genome editing to predict immune checkpoint inhibitor responses. J Hepatol.

[144] Chiu DK (2020). Hepatocellular carcinoma cells up-regulate PVRL1, stabilizing PVR and inhibiting the cytotoxic T-cell response via TIGIT to mediate tumour resistance to PD1 inhibitors in mice. Gastroenterology.

[145] Chuprin J (2023). Humanized mouse models for immuno-oncology research. Nat Rev Clin Oncol.

[146] Kahan B, Levine L (1971). The occurrence of a serum fetal alpha-1 protein in developing mice and murine hepatomas and teratomas. Cancer Res.

[147] Bernhard HP, Darlington GJ, Ruddle FH (1973). Expression of liver phenotypes in cultured mouse hepatoma cells: synthesis and secretion of serum albumin. Dev Biol.

[148] Darlington GJ, Tsai CC, Samuelson LC, Gumucio DL, Meisler MH (1986). Simultaneous expression of salivary and pancreatic amylase genes in cultured mouse hepatoma cells. Mol Cell Biol.

[149] Zabransky DJ (2023). Profiling of syngeneic mouse HCC tumour models as a framework to understand anti-PD-1 sensitive tumour microenvironments. Hepatology.

[150] Kelley RK (2023). Pembrolizumab in combination with gemcitabine and cisplatin compared with gemcitabine and cisplatin alone for patients with advanced biliary tract cancer (KEYNOTE-966): a randomised, double-blind, placebo-controlled, phase 3 trial. Lancet.

[151] Li L (2023). Nanodelivery of scutellarin induces immunogenic cell death for treating hepatocellular carcinoma. Int J Pharm.

[152] Yang X (2021). YIV-906 potentiated anti-PD1 action against hepatocellular carcinoma by enhancing adaptive and innate immunity in the tumour microenvironment. Sci Rep.

[153] Bao X (2021). Enhanced anti-PD-1 therapy in hepatocellular carcinoma by tumour vascular disruption and normalization dependent on combretastatin A4 nanoparticles and DC101. Theranostics.

[154] Shigeta K (2022). Regorafenib combined with PD1 blockade increases CD8 T-cell infiltration by inducing CXCL10 expression in hepatocellular carcinoma. J Immunother Cancer.

[155] Wei CY (2022). PKCalpha/ZFP64/CSF-1 axis resets the tumour microenvironment and fuels anti-PD1 resistance in hepatocellular carcinoma. J Hepatol.

[156] Wu Q (2019). Blocking triggering receptor expressed on myeloid cells-1-positive tumour-associated macrophages induced by hypoxia reverses immunosuppression and anti-programmed cell death ligand 1 resistance in liver cancer. Hepatology.

[157] Guangshun S (2022). Meloxicam inhibits hepatocellular carcinoma progression and enhances the sensitivity of immunotherapy via the MicroRNA-200/PD-L1 pathway. J Oncol.

[158] Wang J (2019). Hepatocellular carcinoma growth retardation and PD-1 blockade therapy potentiation with synthetic high-density lipoprotein. Nano Lett.

[159] Zhu Y (2022). The combination of PD-1 blockade with interferon-alpha has a synergistic effect on hepatocellular carcinoma. Cell Mol Immunol.

[160] Wen L (2019). An efficient combination immunotherapy for primary liver cancer by harmonized activation of innate and adaptive immunity in mice. Hepatology.

[161] Teng CF (2020). Combination therapy with dendritic cell vaccine and programmed death ligand 1 immune checkpoint inhibitor for hepatocellular carcinoma in an orthotopic mouse model. Ther Adv Med Oncol.

[162] Lu X (2023). Combination of AFP vaccine and immune checkpoint inhibitors slows hepatocellular carcinoma progression in preclinical models. J Clin Invest.

[163] Luo Y (2021). Myelocytomatosis-protein arginine n-methyltransferase 5 axis defines the tumourigenesis and immune response in hepatocellular carcinoma. Hepatology.

[164] Shivapurkar N (2023). Treatment with a cholecystokinin receptor antagonist, proglumide, improves efficacy of immune checkpoint antibodies in hepatocellular carcinoma. Int J Mol Sci.

[165] Xiong Z (2023). Targeting PPAR-gamma counteracts tumour adaptation to immune-checkpoint blockade in hepatocellular carcinoma. Gut.

[166] Wang L (2023). Targeting N6-methyladenosine reader YTHDF1 with siRNA boosts antitumour immunity in NASH-HCC by inhibiting EZH2-IL-6 axis. J Hepatol.

[167] Yu M (2022). PARG inhibition limits HCC progression and potentiates the efficacy of immune checkpoint therapy. J Hepatol.

[168] Lin M (2023). Targeting fibrinogen-like protein 1 enhances immunotherapy in hepatocellular carcinoma. J Clin Invest.

[169] Sukowati C (2023). PD-L1 downregulation and DNA methylation inhibition for molecular therapy against cancer stem cells in hepatocellular carcinoma. Int J Mol Sci.

[170] Weng J (2023). Intratumoural PPT1-positive macrophages determine immunosuppressive contexture and immunotherapy response in hepatocellular carcinoma. J Immunother Cancer.

[171] Yang SF (2023). Neoantigen vaccination augments antitumour effects of anti-PD-1 on mouse hepatocellular carcinoma. Cancer Lett.

[172] Zhang L (2023). DBF4 dependent kinase inhibition suppresses hepatocellular carcinoma progression and potentiates anti-programmed cell death-1 therapy. Int J Biol Sci.

[173] Zhu GQ (2023). CD36(+) cancer-associated fibroblasts provide immunosuppressive microenvironment for hepatocellular carcinoma via secretion of macrophage migration inhibitory factor. Cell Discov.

[174] Salman S (2022). HIF inhibitor 32-134D eradicates murine hepatocellular carcinoma in combination with anti-PD1 therapy. J Clin Invest.

[175] Sung PS (2022). Intrahepatic inflammatory IgA(+)PD-L1(high) monocytes in hepatocellular carcinoma development and immunotherapy. J Immunother Cancer.

[176] Wei Y (2021). A FAK inhibitor boosts anti-PD1 immunotherapy in a hepatocellular carcinoma mouse model. Front Pharm.

[177] Yu Z (2022). Nano delivery of simvastatin targets liver sinusoidal endothelial cells to remodel tumour microenvironment for hepatocellular carcinoma. J Nanobiotechnol.

[178] Torrens L (2021). Immunomodulatory effects of lenvatinib plus anti-programmed cell death protein 1 in mice and rationale for patient enrichment in hepatocellular carcinoma. Hepatology.

[179] Yang W (2021). A selective HDAC8 inhibitor potentiates antitumour immunity and efficacy of immune checkpoint blockade in hepatocellular carcinoma. Sci Transl Med.

[180] Yi C (2021). Lenvatinib targets FGF receptor 4 to enhance antitumour immune response of anti-programmed cell death-1 in HCC. Hepatology.

[181] Liu M (2020). Targeting monocyte-intrinsic enhancer reprogramming improves immunotherapy efficacy in hepatocellular carcinoma. Gut.

[182] Shigeta K (2020). Dual programmed death receptor-1 and vascular endothelial growth factor receptor-2 blockade promotes vascular normalization and enhances antitumour immune responses in hepatocellular carcinoma. Hepatology.

[183] Zhu H (2020). Oxaliplatin induces immunogenic cell death in hepatocellular carcinoma cells and synergizes with immune checkpoint blockade therapy. Cell Oncol.

